# Abcès appendiculaires: analyse de 19 cas traités au Centre Hospitalier et Universitaire de Yaoundé et déductions pratiques

**Published:** 2010-07-06

**Authors:** Marc Leroy Guifo, Samuel Takongmo, Alain Chichom, Christopher Tagnyin Pisoh, Faustin Tsatedem Atemkeng, Markus Fokou

**Affiliations:** 1 Faculté de Médecine et des Sciences Biomédicales, Université de Yaoundé I, Cameroun; 2 Faculté des sciences de la santé, Université de Buea, Cameroun; 3 Université de Dschang, Cameroun; 4 Hôpital Général de Yaoundé, Cameroun

**Keywords:** Abcès appendiculaire, voie extra péritonéale, Appendicectomie

## Abstract

**Introduction:**

L’abcès appendiculaire représente 10% des cas d’appendicite aigue. Leur incidence accrue pourrait traduire des difficultés diagnostiques rencontrées en milieux défavorisés ou l’utilisation des examens morphologiques doit être pertinente lorsqu’ils sont disponibles. Le drainage par voie extrapéritonéale si possible suivit d’une appendicectomie à froid était considéré comme l’attitude thérapeutique indiquée. On observe en pratique une incidence faible des appendicectomies différées par rapport à la prévalence des cas d’abcès appendiculaires.

**Méthodes:**

Le but de cette étude était d’évaluer de façon critique la prise en charge des abcès appendiculaires au CHU de Yaoundé. Nous avons rétrospectivement analysé les dossiers de patients traités pour appendicite au CHU de Yaoundé de 2001 à 2006 et les données concernant l’âge, le sexe, les découvertes opératoires, les gestes thérapeutiques étaient recueillis.

**Résultats:**

Dix-neuf cas d’abcès sur 200 cas d’appendicites ont été retrouvés. Dans deux cas seulement il n’y avait pas eu d’appendicectomie au cours de l’intervention soit 10%. On avait 10.5% de complications essentiellement des suppurations pariétales ; comparable à 6% pour les formes non évoluées.

**Conclusion:**

Nous en concluons qu’il est nécessaire de disposer d’un examen morphologique dans les appendicites évoluées avec des manifestations localisées et nous recommandons une prise en charge individualisée.

## Introduction

L’abcès appendiculaire représente 10% des appendicites aigues de l’adulte en Afrique [1]. lI s’agit d’un tableau particulier par le risque de contamination de la cavité péritonéale sous forme d’une péritonite en trois temps et de fistules digestives apres des tentatives d’appendicectomies. Ces risques ont fait accepter comme thérapeutique la pratique d’un drainage par voie extrapéritonéale si possible et une appendicectomie différée. Dans la réalité quotidienne cette attitude conservatrice trouve peu de partisans car on observe une discordance statistique entre les appendicectomies différées et la prévalence des abcès appendiculaires. Le but de l’étude était d’évaluer rétrospectivement le traitement des abcès appendiculaires au CHU de Yaoundé et le confronter aux formes jugées moins évoluées et aux données de la littérature.

## Méthodes

Les registres des hospitalisations et les comptes rendus opératoires ont été revus de janvier 2001 à Décembre 2006 au CHU de Yaoundé. Le service de chirurgie est un service polyvalent dans lequel exercent des enseignants et des chirurgiens de formation diverse. Les patients admis pour appendicite aigue étaient sélectionnés, les dossiers et les comptes rendus opératoires étaient revus. Les données démographiques (âge et sexe), l’échographie préopératoire, la voie d’abord, l’aspect per-opératoire, les gestes thérapeutiques (drainage, appendicectomie), les suites opératoires ainsi que la durée d’hospitalisation étaient analysés. Un total de 200 patients a ainsi été recensé parmi lesquels 19 cas d’abcès appendiculaires.

## Résultats

**Figure 1:**
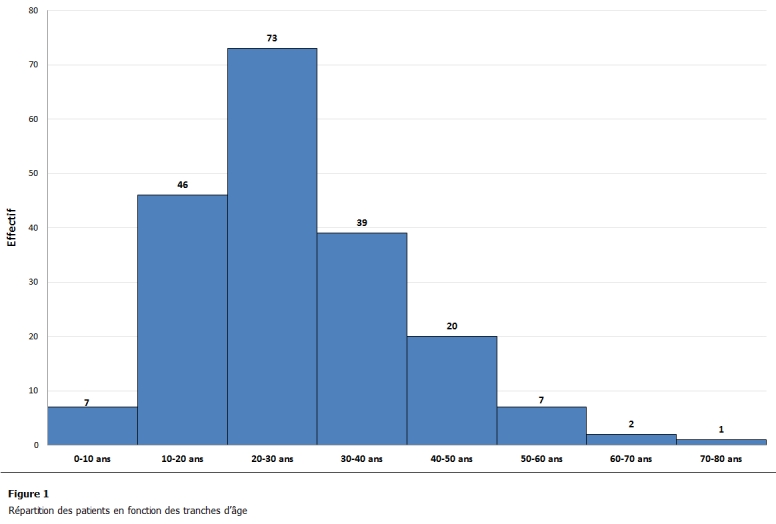
Répartition des patients en fonction des tranches d’âge

Les cas d’appendicite ont été colliges pendant 6 années (N = 200), parmi lesquels 19 cas d’abcès appendiculaires ont été notés (9,5%). L’âge moyen a été de 28 ans pour les cas d’appendicites et 31 ans pour les cas d’abcès (extr 9-75 ans). Le sexe ratio a été de 86 femmes (43%) pour 114 hommes (57%). La répartition des patients par âge a été rapportée (Figure 1).

En fonction des aspects macroscopiques, les lésions étaient qualifiées de catarrhales pour 32 (16%), de congestives ou phlegmoneuses pour 71 (35,5%), de plastron ou de masse pour 9 (4,5%), de gangrenés, perforés ou nécrosés pour 10 (5%), d’abcès pour 19 (9,5%), en péritonite pour 17 (8,5%) et non précisées pour 42 (21%) (Tableau 1).

Les dossiers de 128 patients avaient des informations concernant l’échographie parmi lesquels 79 (39,5%) avait fait l’examen et 49 non (24,5%). Dans 72 (36%) l’information n’était pas précisée. Parmi les 19 cas d’abcès appendiculaire, 12 avaient fait une échographie préopératoire et 7 n’étaient pas précisés. Le scanner n’avait été demandé dans aucun cas.

Pour les voies d’abord ont avait concernant l’abcès appendiculaire 14 abords par une incision au point de Mac Burney, 3 par voie médiane, une voie de Jalaguier et aucune extra péritonéale ou laparoscopique.

**Figure 2:**
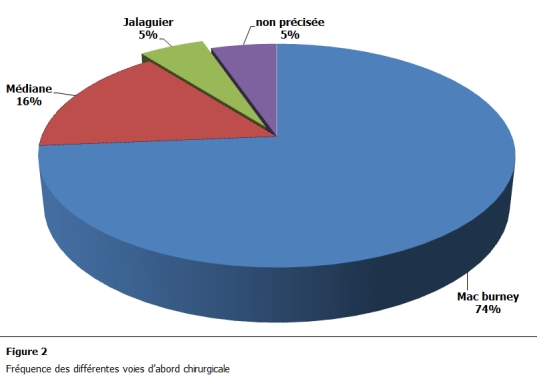
Fréquence des différentes voies d’abord chirurgicale

Au cours de l’opération des cas d’abcès, 14 fois il y a eu appendicectomie pendant l’intervention (73,7%), dans 2 cas elle n’avait pas été faite 10,5% et pour 3 cas cela n’était pas précisé (Figure 2). Les patients n’ayant pas eu une appendicectomie d’emblée ont été opérés à trois mois d’intervalle. Tous les patients présentant un abcès avaient eu un drainage par lame de Delbet à travers une contre incision. Il y’avait eu 2 suppurations pariétales dans les cas d’abcès appendiculaires 10,5% avec un cas ayant présenté à long terme une éventration sur incision de Jalaguier corrigée par plastie. Pour les 181 cas restants on avait un taux de complications de 6 % avec 7cas de suppurations et 5 cas de retard de transit. On a par ailleurs eu 3 décès dont aucun dans le groupe abcès. Une patiente avait une tumeur caecale et avait nécessité secondairement une hémi colectomie droite.

La durée moyenne du séjour hospitalier a été de 8.5 jours pour les cas d’abcès et de 6.2 pour la population d’étude en général avec des extrêmes de 1 et de 23 jours (Tableau 1).

**Tableau 1:** Répartition des patients en fonction de l’aspect anatomopathologique et de la durée de séjour à l’hôpital

	Abcès	Catarrhale	Congestif/phlegmoneux	Plastron/masse	Péritonite/perforé/gangrené/nécrosé	Non précisé	total
Effectif	19	32	71	9	27	42	200
Durée séjour	8,6	5,2	5,5	6,4	8,9		
pourcentage	9,5%	16%	35,5%	4,5%	12.5%	21%	100%

**Figure 3:**
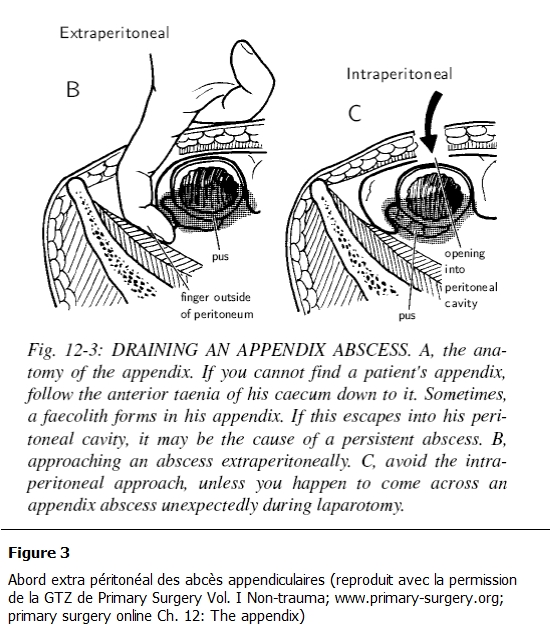
Abord extra péritonéal des abcès appendiculaire (reproduit avec la permission de la GTZ de Primary Surgery Vol. I Non-trauma; www.primarysurgery.org; primary surgery online Ch. 12: The appendix)

## Discussion

Les aspects macroscopiques des appendicites aigues ont été décrits depuis par Jalaguier et nous ont servi de base pour la répartition des patients [2]. Dix-neuf patients sur 200 (9.5%) dans notre étude avaient un abcès appendiculaire. Koumaré et coll sur 109 cas d’appendicites colligés en un an dans les urgences à Bamako trouvent une fréquence de 12.5 % [1]. La stratégie thérapeutique à adopter devant un abcès appendiculaire reste discutée. En 1965 pour G Menegaux "on doit se borner à évacuer le pus par une incision appropriée et ne pas chercher à enlever l’appendice; son ablation étant remise à plus tard une fois l’abcès tari et l’infection complètement disparue (trois à six mois) " [2]. Il s’agissait d’une attitude pédagogique dictée par le souci de la prudence et d’un enseignement homogène. En 2003 dans une série de 30 patients revus rétrospectivement au Nigeria pour masse appendiculaire et comportant 5 cas d’abcès le drainage suivi d’une appendicectomie différée était la stratégie thérapeutique [3]. Il existe cependant des partisans d’une attitude plus agressive ; la prise en charge au CHU de Yaoundé reflète cette position à savoir une appendicectomie dans les cas favorables à la dissection. Elle se justifie parce que le risque ne serait sensiblement pas modifié lors d’une appendicectomie différée et les coûts qui peuvent être considérables pour les patients en général réticents à fréquenter les hôpitaux [4]. On n’a pas observé de différence dans la survenue de suppurations ni de fistules digestives que nous n’avons pas retrouvé dans l’ensemble des cas. Ces résultats sont inférieurs à ceux rapportés dans la littérature à savoir entre 9 et 28 % de complications cumulées [5-7]. La taille de cet échantillon n’est cependant pas représentative.

L’appendicectomie secondaire est aujourd’hui remise en question car certains auteurs pensent qu’après la crise initiale, seuls 25% des patients traités pour masse appendiculaire sans appendicectomie présenteront une récidive. Elle représente pour nous une des explications de la discordance observée par rapport à la fréquence des abcès appendiculaires. Mais nous avons retrouvé 14 cas d’appendicectomie parmi les 19 cas récences (73,7%). Le drainage percutané dans le cas spécifique des abcès et une appendicectomie secondaire représente une attitude conforme aux principes de cette prise en charge mais peu de données spécifiques des milieux défavorisés ou ces tableaux cliniques sont souvent rencontres sont disponibles [8].

Concernant la voie d’abord, l’approche par voie extra péritonéale est recommandée (Figure 3). Elle ne semble pas compatible avec la pratique car la plupart des abcès sont reconnus en peropératoire et de plus peuvent être en position mésocoeliaque ou pelvienne. L’échographie avec une sensibilité de 80% pour le diagnostic peut apporter un complément d’information autant sur le stade anatomopathologique que sur la topographie. Cet examen n’a été pratiqué que pour 40% des patients de notre série. Il pourrait permettre de mieux choisir la voie d’abord. Cette modalité est d’actualité dans les systèmes de santé dans les pays développés où elle se fait à l’aide du Scanner [9]. Une voie transpéritonéale pourrait aussi être indiquée de principe dans les tranches d’âge au-dessus de 40 ans en raison de l’incidence accrus de cancer avec un impératif de biopsie en cas de masse appendiculaire.

La laparoscopie jusqu’ici reste controversée pour ses avantages par rapport à la voie ouverte. Elle est déconseillée par certains auteurs dans les cas évolués notamment les abcès et les appendicites perforées [10,11]. Le taux d’abcès résiduel et de suppuration de paroi retrouvés est variable selon les séries [5,12]. Le drainage systématique tel qu’il a été effectué avait un but prophylactique au cas où il y aurait des complications intra abdominales. Ce choix reste à l’appréciation de l’opérateur et les travaux effectués jusqu’ici n’ont pas abouti à un consensus [10].

## Conclusion

Le diagnostic et le traitement des appendicites aiguës restent variables selon les contextes et les moyens disponibles [13]. Notre étude en étant rétrospective fait une appréciation de la pratique réelle en dehors des courants de pensée. Il apparait ainsi nécessaire de disposer d’un examen morphologique dans les appendicites évoluées avec des manifestations localisées car la reconnaissance de la forme clinique avant l’acte chirurgical a une incidence positive sur les difficultés et les suites opératoires. L’appendicectomie d’emblée peut être pratiquée pour les abcès appendiculaires de découverte peropératoire lorsque la dissection parait possible et la voie d’abord adaptée. Nous recommandons cependant des études actualisées, prospectives et plus larges pour une meilleure évaluation.

## Conflits d’intérêts

Les auteurs ne déclarent aucun conflit d’intérêts. Ce travail a été présenté à la réunion annuelle du Collège Américain de Chirurgie en 2007 au cours de la séance réservée aux lauréats de l'IGS (International Schorlar Guest).

## Contribution des auteurs

MG a fait la collecte des données et la rédaction du manuscrit. ST a coordonné l’étude, contribuer à la révision du manuscrit et approuvé la version finale. AC a lu le manuscrit et fait des suggestions. CP a pris en charge des cas. FA et MF ont participé à la collecte et à l’analyse des données et la rédaction du manuscrit.
